# Sub-nanometer ultrathin epitaxy of AlGaN and its application in efficient doping

**DOI:** 10.1038/s41377-022-00753-4

**Published:** 2022-03-24

**Authors:** Jiaming Wang, Mingxing Wang, Fujun Xu, Baiyin Liu, Jing Lang, Na Zhang, Xiangning Kang, Zhixin Qin, Xuelin Yang, Xinqiang Wang, Weikun Ge, Bo Shen

**Affiliations:** 1 State Key Laboratory of Artificial Microstructure and Mesoscopic Physics, School of Physics, Peking University, 100871 Beijing, China; 2 Nano-optoelectronics Frontier Center of Ministry of Education, Peking University, 100871 Beijing, China; 3 Collaborative Innovation Center of Quantum Matter, 100871 Beijing, China

**Keywords:** Inorganic LEDs, Electronics, photonics and device physics

## Abstract

Solving the doping asymmetry issue in wide-gap semiconductors is a key difficulty and long-standing challenge for device applications. Here, a desorption-tailoring strategy is proposed to juggle the carrier concentration and transport. Specific to the p-doping issue in Al-rich AlGaN, self-assembled p-AlGaN superlattices with an average Al composition of over 50% are prepared by adopting this approach. The hole concentration as high as 8.1 × 10^18^ cm^−3^ is thus realized at room temperature, which is attributed to the significant reduction of effective Mg activation energy to 17.5 meV through modulating the activating path, as well as the highlighted Mg surface-incorporation by an intentional interruption for desorption. More importantly, benefiting from the constant ultrathin barrier thickness of only three monolayers via this approach, vertical miniband transport of holes is verified in the p-AlGaN superlattices, greatly satisfying the demand of hole injection in device application. 280 nm deep-ultraviolet light-emitting diodes are then fabricated as a demo with the desorption-tailored Al-rich p-AlGaN superlattices, which exhibit a great improvement of the carrier injection efficiency and light extraction efficiency, thus leading to a 55.7% increase of the light output power. This study provides a solution for p-type doping of Al-rich AlGaN, and also sheds light on solving the doping asymmetry issue in general for wide-gap semiconductors.

## Abstract (web summary)

A desorption-tailoring strategy is demonstrated to steadily prepare self-assembled p-AlGaN superlattices with sub-nanometer ultrathin barriers by MOCVD, which juggle the hole concentration and transport.

## Graphical abstract

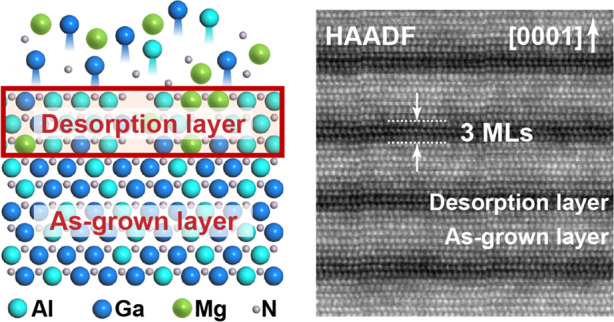

## Introduction

Semiconductors stand at the heart of modern electronics and optoelectronics, which is largely attributed to the development of controllable doping techniques. Although some devices can be realized with unipolar conduction^1–3^, the majority hinge on having both high-quality n- and p-type doping^4–6^. Unfortunately, semiconductors, especially wide-gap semiconductors, suffer from a long-standing doping asymmetry issue, i.e., one type of doping is achieved rather easily, while the other is turned out to be an enormous challenge^7^. Taking ZnO and AlGaN as examples, they all have preferable n-type conductivity, but it requires immense efforts to achieve p-type one since their valence bands are relatively far from the vacuum level^8,9^. The case is contrary for diamond^10^. The doping asymmetry issue severely restricts the development of devices, typically AlGaN-based deep-ultraviolet optoelectronic devices, which are subjected to the lack of high-quality Al-rich p-AlGaN^11^.

When attempting to solve the doping issue, carrier concentration and transport must be juggled. In terms of the carrier concentration, there are several scenarios that need to be considered, including (i) solubility of dopants, (ii) dopants being on the right lattice positions, (iii) activation energy of dopants, and (iv) self-compensation by intrinsic defects. For carrier transport, efforts should be devoted to satisfying the demand for carrier injection in device applications. Still taking Al-rich p-AlGaN as an example, although Mg is found to be a relatively successful p-type dopant, the essential difficulties persist, especially exceptionally high acceptor activation energy and quite a low solubility of Mg. In III-nitrides, the activation energy of Mg increases from 160 meV in GaN to as high as 510 meV in AlN^12^. Hence the activation energy of higher than 300 meV can be expected in Al-rich (>50%) AlGaN, which is far greater than the thermal energy of 26 meV at room temperature, and thus resulting in an extremely low activation efficiency of Mg. What is worse, due to the positive and large formation enthalpies, the solubility of Mg as a substitute for Ga or Al in AlGaN is quite limited^13^. On account of the above two factors, the hole concentration in Al-rich p-AlGaN is terribly low, far from satisfying the demand of devices. A number of targeted approaches have been proposed, including superlattice (SL) doping, quantum-dots doping, and polarization-induced doping for reducing the activation energy of Mg^14–16^; delta doping and modified surface engineering for enhancing Mg incorporation^13,17^, etc. Although some approaches have been employed to fabricate related devices^18–20^, there is still a lack of a thorough solution for p-type doping in Al-rich AlGaN. Most approaches cannot cover all considerations above, or even cause negative effects in some aspects when solving the others. For example, SL doping effectively reduces the activation energy by modulating the band structure, the vertical hole transport is however usually blocked by the barriers, particularly since it is quite difficult to stably obtain ultrathin (less than 1 nm) ternary AlGaN. Similarly, modified surface engineering significantly enhances the Mg incorporation into AlGaN by creating ultimate N-rich conditions, but the trouble of high activation energy persists. Therefore, an all-round approach to solve the p-doping issue in Al-rich AlGaN is eagerly expected.

In this study, we propose a desorption-tailoring strategy to juggle the carrier concentration and transport. Self-assembled p-AlGaN SLs with an average Al composition of 51% is prepared by this approach, in which the effective activation energy of Mg acceptors is greatly reduced due to the modulation of the hole activating path; meanwhile, the Mg concentration in AlGaN is significantly increased, benefiting from the highlighted surface-incorporation effect by intentional interruptions for desorption. As such, the hole concentration as high as 8.1 × 10^18^ cm^−3^ is achieved at room temperature. More significantly, beneficial from the constant ultrathin barrier thickness of 3 monolayers (MLs) in this approach, vertical miniband transport of holes is verified in the p-AlGaN SLs, greatly satisfying the demand of hole injection in devices. As an application, 280 nm deep-ultraviolet light-emitting diodes (DUV-LEDs) are fabricated with the desorption-tailored Al-rich p-AlGaN SLs, which then exhibit a great improvement of carrier injection efficiency and light extraction efficiency.

## Results

**Fig. 1:**
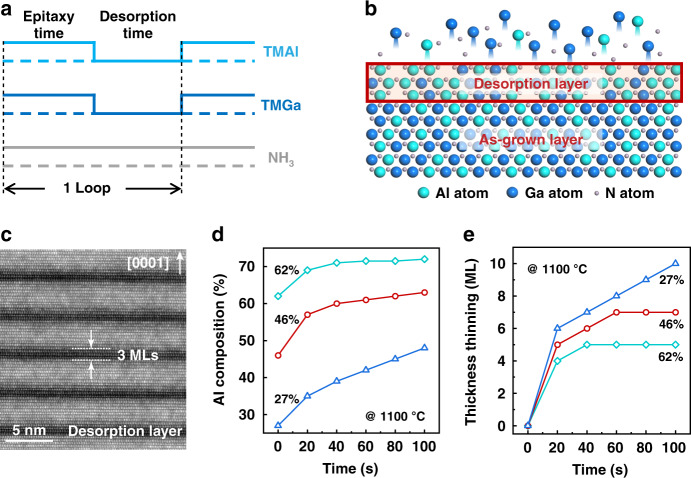
Schematic process and corresponding structural features of the interrupted epitaxy. **a** Schematic process of the periodically interrupted epitaxy. **b** Schematic illustration of the self-assembled bilayer structure by desorption-tailoring. **c** Z-contrast image of the self-assembled bilayer structures in AlGaN with the desorption time and initial Al composition of 80 s (at 1100 °C) and 46%, respectively. **d** Desorption time dependence of Al composition in the desorption layer. **e** Desorption time dependence of the thinning thickness in the as-grown layer

Periodically interrupted epitaxy is carried out to prepare AlGaN SLs as shown in Fig. 1. Generally, epitaxy is dominated by two major processes: one is the adsorption of atoms to the surface, which is followed by their diffusion and incorporation into lattices; while the other is the reverse desorption process, namely breaking-away of atoms from lattices as well as their desorption from the surface. When the adsorption/incorporation rate is higher than the desorption one, epitaxy occurs (epitaxy time in Fig. 1a). However, if the supply of the precursors is intentionally interrupted, (desorption time as shown in Fig. 1a, other growth conditions unchanged) the adsorption and incorporation process is considered to be suspended, meanwhile desorption persists and dominates. Specific to the interrupted epitaxy of AlGaN, a homogeneous Al composition is achieved with the fluxes of III-precursors being fixed in the epitaxial time, while during the intentional interruption, an increase of Al composition on the surface is expected since there is a higher desorption rate of Ga atoms than Al ones owing to the lower binding energy of GaN^21^. Moreover, three associated structural features in the interrupted epitaxy are experimentally observed in Fig. 1c–e, including:

(i) Desorption-tailored bilayer structure and constant ultrathin thickness of the desorption layer. A self-assembled bilayer structure with a smooth and sharp interface can be observed as shown in Fig. 1c, where the desorption time and initial Al composition are 80 s (at 1100 °C) and 46%, respectively. It is demonstrated that the layers with deep or shallow contrast in Fig. 1c correspond to the desorption and as-grown layer (Fig. 1b), respectively. (Fig. S1, supplementary information) The thickness of the desorption layers maintains at 3 MLs (about 0.75 nm for AlGaN), which is verified to remain constant in a larger range of initial Al composition (15–80%), desorption temperature (1040–1160 °C), and desorption time (20–100 s).

(ii) Increase of Al composition in the desorption layer. The contrast difference in Fig. 1c indicates higher Al composition in the desorption layer than that in the as-grown layer, which is attributed to the higher desorption rate of Ga atoms as mentioned above. Moreover, Al composition in the desorption layer depends directly on the desorption time as shown in Fig. 1d, where Al composition increases continuously with time.

(iii) Thickness thinning of the as-grown layer. On account of the desorption and rearrangement of surface atoms, it is rational that the as-grown layer will be thinned during the desorption process. Figure 1e shows the desorption time dependence of the thinning thickness in the as-grown layer at 1100 °C. Fast thinning is observed within the initial 20 s, which corresponds to the formation of the desorption layer. When further prolonging the desorption time, the thinning process gradually slows down.

Thereby a desorption-tailoring strategy based on the interrupted epitaxy is proposed, where SLs of specified structure can be prepared. On the one hand, by adjusting the desorption time, the composition difference between well and barrier in SLs can be finely regulated; on the other hand, by matching the desorption time and initial thickness of the as-grown layer (epitaxy time), the thickness of the well layers in SLs can be effectively controlled, while keeping the barrier layers to be 3 MLs.

**Fig. 2:**
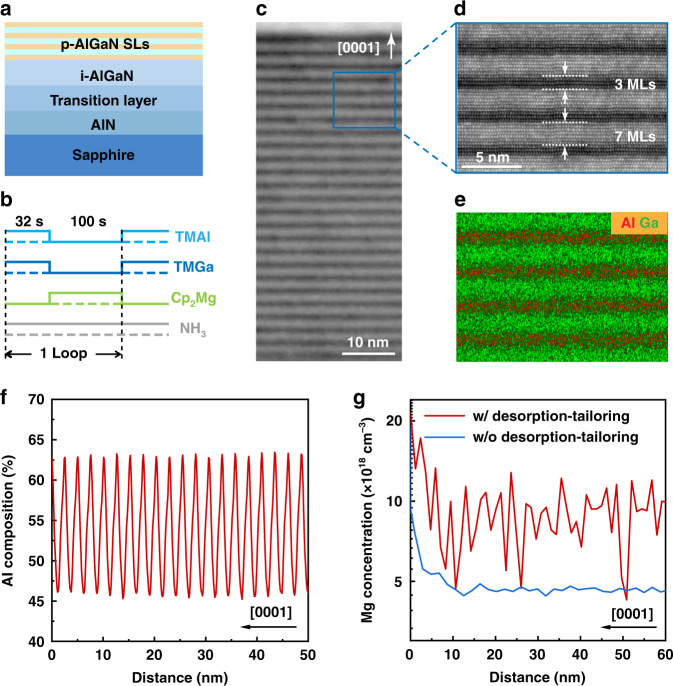
Preparation of Al-rich p-AlGaN SLs by adopting the desorption-tailoring strategy. **a** Schematic illustration of the sample with desorption-tailored p-AlGaN SLs. **b** Schematic process of desorption-tailoring for p-AlGaN SLs. **c** HRTEM image of the p-AlGaN SLs. **d** Z-contrast image of the p-AlGaN SLs. **e** Distribution of Al and Ga atoms by EDS mapping corresponding to the region in panel **d**. **f** EDS line scanning curve of Al composition in the p-AlGaN SLs. **g** Mg concentration profile by SIMS in the desorption-tailored p-AlGaN SLs as well as in an uninterrupted-grown Al_0.63_Ga_0.37_N epilayer (without desorption-tailoring)

The approach is then adopted to prepare p-AlGaN SLs (Fig. 2a) with the epitaxy and desorption time to be 32 and 100 s, respectively. The thickness of the barrier and well layers is then well determined to be 3 and 7 MLs, respectively, as shown in the Z-contrast image of Fig. 2d. Moreover, obvious periodic atomic distribution of Al and Ga can be observed by energy-dispersive X-ray spectroscopy (EDS) mapping (Fig. 2e), which corresponds to Al composition of about 63% and 46% for the barrier and well, respectively (Fig. 2f). The average Al composition in the self-assembled p-AlGaN SLs is thus calculated to be 51%.

For Mg incorporation, the Mg dopants are introduced just during the desorption process as shown in Fig. 2b. On the one hand, Mg atoms can naturally occupy the “right” III-sites in the lattices, following the desorption of Ga and Al atoms; on the other hand, the suspending of III-precursors highlights the surface-incorporation effect by producing an extremely N-rich condition, which modulates the formation enthalpy of Mg_Ga_ and Mg_Al_ to negative values, and thus enhances Mg incorporation into AlGaN^13^. The barriers in the SLs can then be effectively Mg-doped, while the wells nominally undoped^22^, in consistent with the periodic oscillation of Mg concentration (the red line) by secondary ion mass spectroscopy (SIMS) as shown in Fig. 2g. Peaks in SIMS indicate that the Mg concentration in the Al_0.63_Ga_0.37_N barriers is over 1 × 10^19^ cm^−3^, about one time higher than that in a referential Al_0.63_Ga_0.37_N epilayer (continuously grown, without desorption) with the same Cp_2_Mg flux (the blue line).

**Fig. 3:**
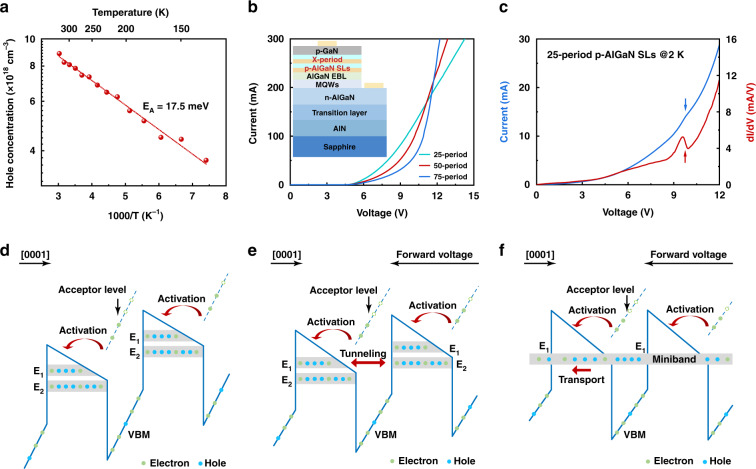
Electrical properties of the desorption-tailored Al-rich p-AlGaN SLs. **a** Temperature dependence of the hole concentration in the desorption-tailored p-AlGaN SLs. **b** I–V curves at room temperature of DUV-LED structures (inset) with p-AlGaN SLs period of 25, 50, and 75, respectively. **c** I–V and corresponding dI/dV curves at 2 K of DUV-LED structure with 25-period p-AlGaN SLs. **d** The upward inclining of the p-AlGaN SLs energy band profile along [0001] direction at equilibrium. **e** Resonant tunneling between E_1_ and adjacent E_2_ when flattening the p-AlGaN SLs energy band profile along [0001] direction by applying a forward voltage. **f** Formation of minibands when applying a higher forward voltage

Electrical properties of the desorption-tailored Al-rich p-AlGaN SLs are then characterized, including the hole concentration and vertical transport. The hole concentration is determined to be as high as 8.1 × 10^18^ cm^−3^ at room temperature by Hall effect measurements, which is one of the highest values for Mg-doped Al-rich AlGaN reported to date^14,15,23–25^. (Table S1, supplementary information) Moreover, the hole concentration exhibits a weak temperature dependence as shown in Fig. 3a. (temperature dependence of mobility shown in Fig. S2, supplementary information) By employing the Arrhenius equation, the effective activation energy of Mg in the p-AlGaN SLs is extracted to be 17.5 meV, much lower than the thermal energy of 26 meV at room temperature, indicating that the desorption-tailored p-AlGaN SLs effectively overcome the prominent difficulty of high activation energy of Mg in Al-rich AlGaN. It is inferred that the reduction of the effective activation energy is attributed to the modulation of the hole activation path as schematically shown in Fig. 3d. Owing to the polarization-induced band bending in p-AlGaN SLs, the acceptor level in the barriers is quite close to the sub-band level in the wells, which then provides an energetically favorable path for the activation of Mg.

Furthermore, the vertical hole transport in the desorption-tailored p-AlGaN SLs is demonstrated, for which a series of samples with different periods (25, 50, and 75) of SLs are prepared with the structure shown in the insert of Fig. 3b. Figure 3b shows the forward I–V curves of the three samples at room temperature, where the turn-on voltages are almost the same (about 5 V), indicating good consistency for the sample growth. As the voltage increases from 5 V, the three samples present quite different I–V characteristics. The sample with more periods has a lower slope at the beginning, i.e., higher voltage is required for the same current; while after a turning value of voltage (9, 10, and 11 V for 25, 50, and 75 periods, respectively), the slopes increase sharply, and the sample with more periods has a greater slope. The resistance-like characteristic of p-AlGaN SLs before the turning point is supposed to be related to the polarization-induced energy band inclining as shown in Fig. 3d, which is caused by the different polarization charge densities at the top (p-AlGaN SLs/p-GaN) and bottom (EBL/p-AlGaN SLs) interfaces of the p-AlGaN SLs (Fig. S5). The upward inclining along [0001] direction inhibits the formation of minibands until being counteracted by a forward voltage as shown in Fig. 3f. Considering the polarization field is the same for the three samples, it is rational that the sample with more periods requires a higher forward voltage to flatten the energy band, well consistent with the higher voltage before the turning point.

After the voltage turning point, the difference of the slopes demonstrates a typical feature of miniband transport in the SLs. By derivative analysis of the linear rising segment in I–V curves^26^, series resistances of the three samples can be extracted to be 22.7, 11.9, and 3.8 Ω for 25, 50, and 75 periods, respectively. The inverse relationship between series resistance and SLs period (thickness) indicates a true miniband transport in SLs, where the miniband width plays a leading role. As reported by R. Tsu and L. Esaki^27^, the stronger the coupling between adjacent wells and the more periods in finite SLs, the wider the miniband width would be. For the desorption-tailored SLs, the coupling between the adjacent wells is guaranteed by the ultrathin barrier of 3 MLs, which is invariant for the three samples. Hence the period directly determines the width of the miniband, i.e., 75 periods SLs are expected to have the wider miniband width, implying higher mobility of vertical hole transport as well as smaller series resistance^28^, in consistence with the results in Fig. 3b. By analyzing the I–V characteristics, it is convinced that the miniband does exist in the desorption-tailored p-AlGaN SLs, which provides an effective way for vertical hole transport.

In order to further study the vertical hole transport, I–V measurements at cryogenic temperature (2 K) are carried out. Figure 3c shows the I–V and corresponding dI/dV curves of the sample with 25-period p-AlGaN SLs. The feature of the negative differential resistance can be observed at about 9 V as indicated by the arrow^29^, which is absent when only replacing the p-AlGaN SLs with thick p-GaN. It is supposed that the resonant tunneling occurs^29^ firstly between E_1_ and the adjacent E_2_ level (Fig. 3e) in the process of flattening the energy band by applying a forward voltage; with further increase of voltage, the resonant tunneling is destroyed and then re-established between E_1_ levels in adjacent wells (Fig. 3f), leading to the negative differential resistance as observed. The realization of resonant tunneling provides the crucial foundation for the formation of minibands in the p-AlGaN SLs^28^.

**Fig. 4:**
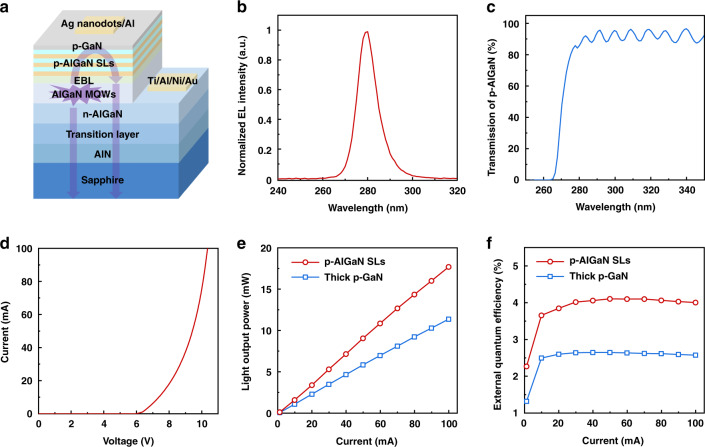
Performance of DUV-LEDs fabricated with desorption-tailored Al-rich p-AlGaN SLs. **a** Schematic illustration of DUV-LEDs with desorption-tailored p-AlGaN SLs. **b** EL spectrum (at 100 mA) of DUV-LEDs. **c** Transmission spectrum of the desorption-tailored p-AlGaN SLs (without the p-GaN contact layer). **d** I–V curve of DUV-LEDs with the p-electrode of complex Ag nanodots/Al. **e, f** Dependence of the LOP and EQE on the injection current for DUV-LEDs with desorption-tailored p-AlGaN SLs and thick p-GaN, respectively

As an application, 280 nm DUV-LEDs are fabricated with the desorption-tailored p-AlGaN SLs as shown in Fig. 4a, b, where two advantages can show their real effects. For one thing, the Al-rich p-AlGaN SLs are beneficial to hole injection, which is attributed to the high hole concentration and unhindered vertical hole transport; for another, it lays the foundation for extracting the light emitted towards p-type region, since the p-AlGaN SLs (without the p-GaN contact layer) is transparent to the light with wavelength of 280 nm as shown in Fig. 4c. Then complex Ag nanodots/Al is adopted as the highly reflective p-electrode^30^, and 8 nm-thick p-GaN acts as the contact layer to ensure good Ohmic contact. DUV-LEDs show good rectifying behavior with a turn-on voltage of 6.2 V in the I–V curve as shown in Fig. 4d.

Figure 4e shows the dependence of the light output power (LOP) on the injection current after encapsulation with a hemisphere lens. Reference devices are fabricated simultaneously by replacing the p-AlGaN SLs with p-GaN of the same thickness. (Fig. S3, supplementary information) It is found that the LOP of DUV-LEDs with p-AlGaN SLs reaches 17.7 mW at 100 mA, 55.7% better than that with thick p-GaN. Thus the external quantum efficiency (EQE) is calculated to be 4.1% in DUV-LEDs with p-AlGaN SLs (Fig. 4f). Through comparative analysis, the enhancement of the light extracting efficiency (LEE) contributes about two-thirds of the performance improvement, while the rest is from the enhancement of the carrier injection efficiency (CIE). (Fig. S4, supplementary information).

## Discussion

In summary, a desorption-tailoring strategy is proposed to overcome the enormous challenge of doping asymmetry, in which the carrier concentration and transport are juggled. Applying that to p-AlGaN, self-assembled p-AlGaN SLs with an average Al composition of 51% are conducted. Based on the significant reduction of the effective acceptor activation energy as well as the substantial enhancement of Mg surface-incorporation, the hole concentration as high as 8.1 × 10^18^ cm^−3^ is realized at room temperature. More importantly, beneficial from the constant ultrathin barrier thickness of 3 MLs by this approach, the vertical miniband transport of holes is verified in the p-AlGaN SLs, greatly satisfying the demand of hole injection in devices. As an application, 280 nm DUV-LEDs are fabricated with the desorption-tailored Al-rich p-AlGaN SLs, which lead to a 55.7% increase of the LOP. It is expected that this study provides a solution for p-doping in Al-rich AlGaN, and it sheds light on solving the doping asymmetry issue in general for wide-gap semiconductors, especially for ternary and quaternary compound semiconductors.

## Materials and methods

### Preparation of samples by MOCVD

All samples in this study were grown by a 3 × 2′′ close-coupled-showerhead (CCS) MOCVD system. For the desorption-tailored p-AlGaN SLs, 1 μm-thick AlN was grown on (0001) sapphire at 1220 °C, followed by 20 pairs of AlN/Al_0.8_Ga_0.2_N and 20 pairs of Al_0.8_Ga_0.2_N /Al_0.6_Ga_0.4_N as the transition layer. Then, 200 nm-thick undoped Al_0.6_Ga_0.4_N was grown, on which the periodically interrupted epitaxy is adopted to prepare the p-AlGaN SLs. For accurate electrical measurement, 8 nm-thick p-GaN was grown on the top as a contact layer for Ohmic contact, which was then etched except the area below the p-electrode.

### Fabrication of DUV-LEDs

The DUV-LED structure was grown on sapphires, with AlN and transition layer the same as above. Then, 1.5 μm-thick n-Al_0.55_Ga_0.45_N, 5-period Al_0.5_Ga_0.5_N/Al_0.37_Ga_0.63_N multiple quantum wells (MQWs), 10 nm-thick p-Al_0.8_Ga_0.2_N electron blocking layer (EBL), desorption-tailored 50-period p-Al_0.46_Ga_0.54_N/Al_0.63_Ga_0.37_N SLs, and 8 nm-thick p-GaN contact layer were grown in sequence. DUV-LED devices with a die size of 889 × 889 μm^2^ were fabricated with the standard flip-chip process. A Ti/Al/Ni/Au metal stack was deposited on the exposed n-AlGaN as the n-electrode, and a complex Ag nanodots/Al stack was used as the highly reflective p-electrode.

### Characterizations and measurements

The periodically interrupted epitaxy for AlGaN SLs was characterized with STEM (Thermo Scientific Themis Z, operating at 200 kV). The specimens for STEM were prepared by FIB (Thermo Scientific Helios G4 HX) with a thickness of about 50 nm. SIMS (Cameca IMS-6F), Hall effect (Accent HL5550 LN2), and I–V (Keithley 2400) were carried out. Transmission spectrum is measured by Varian Cary 5000 UV-Vis spectrophotometry, and a reference sample containing all structures except for the p-AlGaN SLs is used to account for the influences of other layers on the measurements. For I–V at cryogenic temperature, physical property measurement system (PPMS, Quantum Design) was used. The LOP of DUV-LEDs was measured by an integrating sphere for UV light (Everfine).

## Supplementary information

Supplementary information

## Data Availability

The data that support the findings of this study are available from the corresponding authors upon reasonable request.
