# Supplementary material for: Sub-nanometer ultrathin epitaxy of AlGaN and its application in efficient doping
Source: Light Sci Appl. 2022 Mar 24;11:71. doi: 10.1038/s41377-022-00753-4 (PMC8943166; doi:10.1038/s41377-022-00753-4)
Supplement: Supplementary file 1 — Supplementary information [file 41377_2022_753_MOESM1_ESM.docx]

**Supplementary information**

**Sub-nanometer ultrathin epitaxy of AlGaN and its application in efficient doping**

Jiaming Wang,^1^ Mingxing Wang,^1^ Fujun Xu,^1,*^ Baiyin Liu,^1^ Jing Lang,^1^ Na Zhang,^1^ Xiangning Kang,^1^ Zhixin Qin,^1^ Xuelin Yang,^1^ Xinqiang Wang,^1,2,3^ Weikun Ge,^1^ and Bo Shen^1,2,3,**^

^1^State Key Laboratory of Artificial Microstructure and Mesoscopic Physics, School of Physics, Peking University, Beijing 100871, China

^2^Nano-optoelectronics Frontier Center of Ministry of Education, Peking University, Beijing 100871, China

^3^Collaborative Innovation Center of Quantum Matter, Beijing 100871, China

Correspondence: Fujun Xu (fjxu@pku.edu.cn) or Bo Shen (bshen@pku.edu.cn).

**I. Identification of the desoption/as-grown layer in the desorption-tailored bilayer structures**


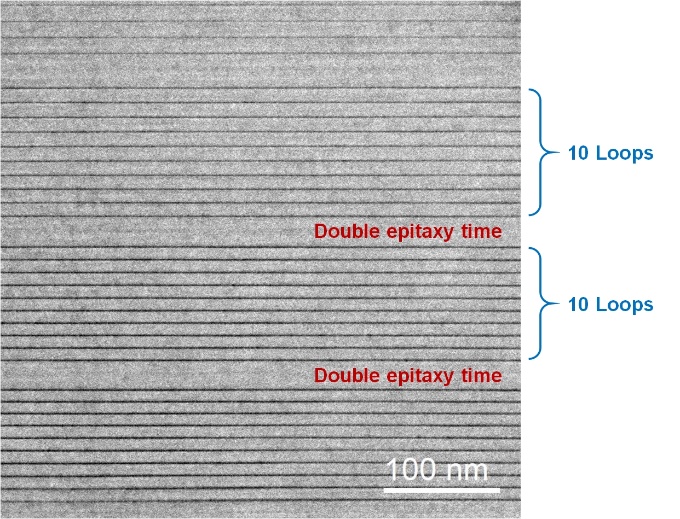


**Fig. S1** HAADF image for the clarification of structural features in the desorption-tailoring approach. In order to identify the the desoption/as-grown layer in the desorption-tailored bilayer structures, the epitaxy time after every 10 loops is intentionally doubled, which suggest a significantly thickened as shown in the HAADF image. And thus we can distinguish the layers by contrast, namely the desorption layers (dark) with deep contrast and the as-grown layers (light) with shallow contrast. Also, it is found that the Al composition in the desorption layers is higher than that in the as-grown ones.

**II. Hole concentration in Mg-doped Al-rich AlGaN**

Table S1. Hole concentration reported in Mg-doped Al-rich p-AlGaN

| Approach | Average Al composition (%) | Hole concentration (cm^-3^) | Reference |
| --- | --- | --- | --- |
| Co-doping | 40 | 6×10^18^ | *Appl. Phys. Lett.* **99**, 112110 (2011)^[23]^ |
| Mg-delta doping | 42 | 8.3×10^18^ | *Mater. Adv.* **1**, 77 (2020)^[24]^ |
| SLs doping | 58 | 3.5×10^18^ | *Sci. Rep.* **6**, 21897 (2016)^[25]^ |
| QDs doping | 60 | 1.25×10^18^ | *Light: Sci. Appl.* **10**, 69 (2021)^[15]^ |
| SLs doping | 81 | 1×10^18^ | *Jpn. J. Appl. Phys.* **57**, 04FH09 (2018)^[14]^ |
| SLs doping | 51 | 8.1×10^18^ | This work |

**III. Temperature-dependent hole mobility in the desorption-tailored p-AlGaN SLs**


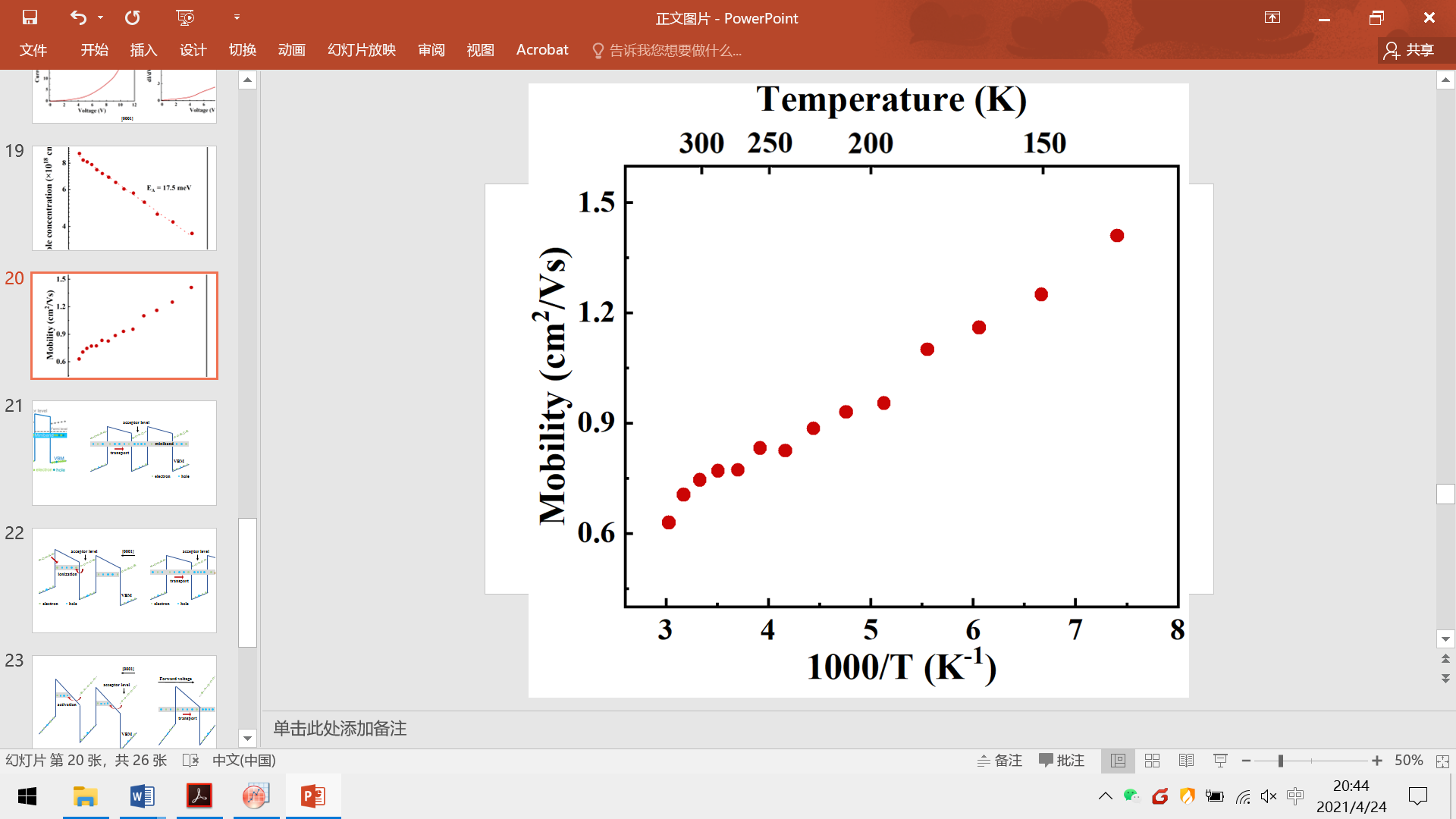


**Fig. S2** Temperature dependence of the hole mobility in the desorption-tailored p-AlGaN SLs measured by Hall effect.

**IV. Comparison between DUV-LEDs with desorption-tailored p-AlGaN SLs and thick p-GaN**

**
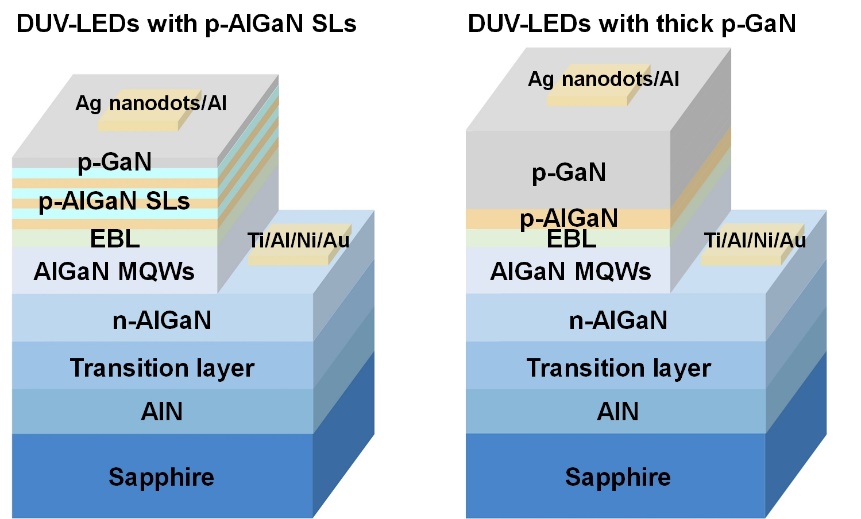
**

**Fig. S3** Schematic illustration of DUV-LEDs with desorption-tailored p-AlGaN SLs (the validation device) and thick p-GaN (the reference device), respectively. The main difference between the two samples is the 50-period p-AlGaN SLs (about 125 nm) in the validation DUV-LED are replaced by p-GaN of the same thickness in the reference one, acting as the p-cladding layer. Also, a thin Al_0.6_Ga_0.4_N layer (about 30 nm) between EBL and p-GaN is grown in the reference device, which is nominally p-doped. Other layers, as well as the fabrication process, are exactly the same between these two devices.

**
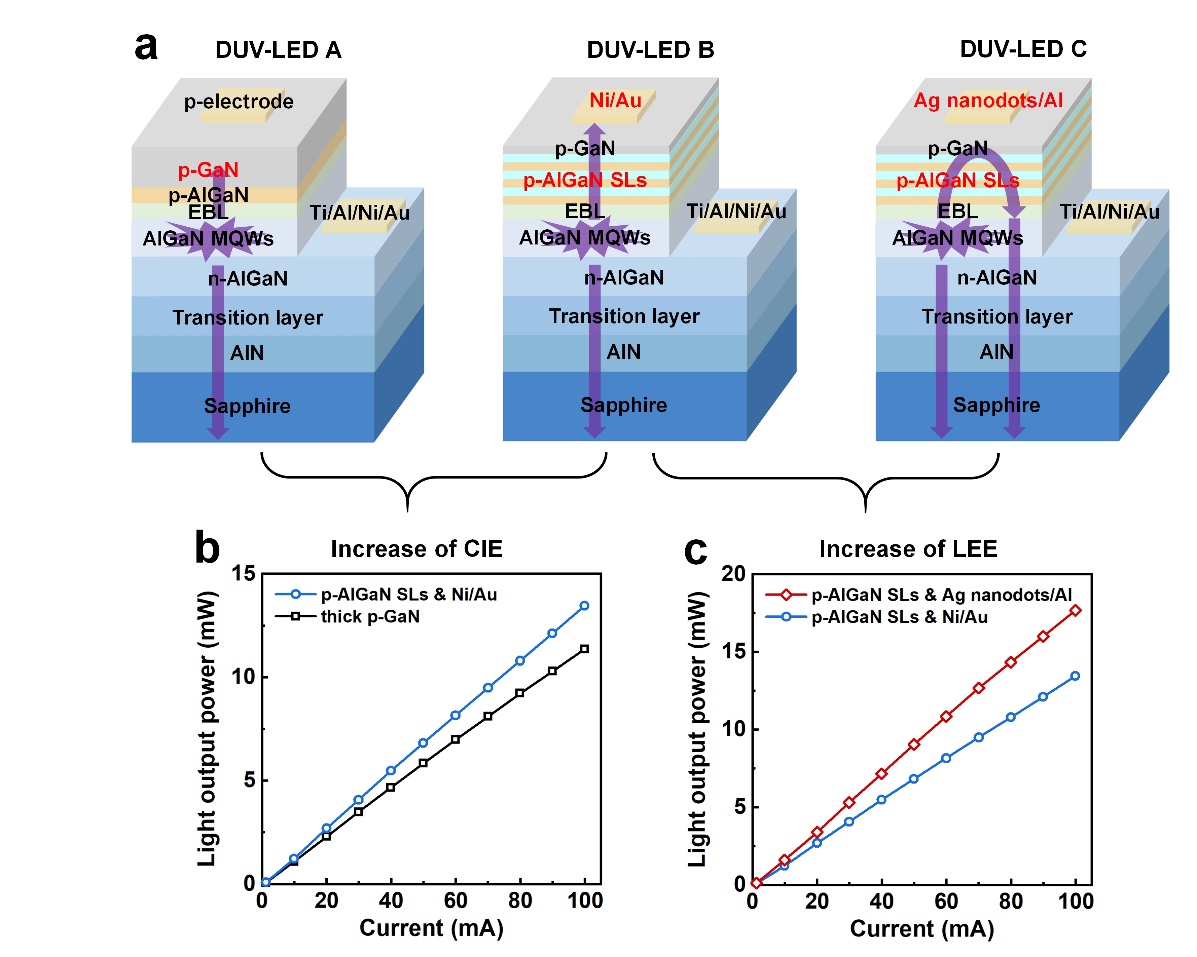
**

**Fig. S4** Since the active region (AlGaN MQWs) is unchanged in this study, the performance improvement of DUV-LEDs is attributed to the enhancement of CIE and LEE. In order to distinguish them, another reference DUV-LED (B here) is prepared in addition to the two DUV-LEDs (A & C) described in Fig. S3.

**(i) Enhancement of CIE**. Considering that the thick p-GaN (about 125 nm in DUV-LED A) can absorb the overwhelming majority of the 280-nm light emitted towards p-type region, Ni/Au is intentionally adopted as the p-electrode in DUV-LED B, which absorbs most of the 280-nm light emitted towards p-type region and then largely eliminates the LEE difference between DUV-LED A and B. An apparent increase of the LOP (2.10 mW at 100 mA) is observed in DUV-LED B as shown in Fig. S4b, which is then attributed to the enhancement of the CIE.

**(ii) Enhancement of LEE.** Ni/Au and Ag nanodots/Al are adopted as the p-electrode in DUV-LED B and C, respectively, whose epitaxial structures are exactly the same to eliminate the CIE difference. There is a significant increase of the LOP (4.22 mW at 100 mA) in DUV-LED C as shown in Fig. S4c, which can be attributed to the enhancement of the LEE.

In conclusion, the performance improvement of DUV-LEDs with p-AlGaN SLs is attributed to the enhancement of the CIE and LEE, among them the enhancement of the LEE contributes about two-thirds, while the rest from the CIE.
